# Rapid L2 Word Learning through High Constraint Sentence Context: An Event-Related Potential Study

**DOI:** 10.3389/fpsyg.2017.02285

**Published:** 2017-12-22

**Authors:** Baoguo Chen, Tengfei Ma, Lijuan Liang, Huanhuan Liu

**Affiliations:** ^1^Beijing Key Laboratory of Applied Experimental Psychology, Faculty of Psychology, National Demonstration Center for Experimental Psychology Education, Beijing Normal University, Beijing, China; ^2^School of Education, Open University of China, Beijing, China; ^3^Bilingual Cognition and Development Lab, School of English and Education, Guangdong University of Foreign Studies, Guangzhou, China; ^4^Research Center of Brain and Cognitive Neuroscience, Liaoning Normal University, Dalian, China

**Keywords:** second language, contextual word learning, sentence constraint, presentation order, proficiency

## Abstract

Previous studies have found quantity of exposure, i.e., frequency of exposure (Horst et al., 1998; Webb, 2008; Pellicer-Sánchez and Schmitt, 2010), is important for second language (L2) contextual word learning. Besides this factor, context constraint and L2 proficiency level have also been found to affect contextual word learning (Pulido, 2003; Tekmen and Daloglu, 2006; Elgort et al., 2015; Ma et al., 2015). In the present study, we adopted the event-related potential (ERP) technique and chose high constraint sentences as reading materials to further explore the effects of quantity of exposure and proficiency on L2 contextual word learning. Participants were Chinese learners of English with different English proficiency levels. For each novel word, there were four high constraint sentences with the critical word at the end of the sentence. Learners read sentences and made semantic relatedness judgment afterwards, with ERPs recorded. Results showed that in the high constraint condition where each pseudoword was embedded in four sentences with consistent meaning, N400 amplitude upon this pseudoword decreased significantly as learners read the first two sentences. High proficiency learners responded faster in the semantic relatedness judgment task. These results suggest that in high quality sentence contexts, L2 learners could rapidly acquire word meaning without multiple exposures, and L2 proficiency facilitated this learning process.

## Introduction

The accumulation of vocabulary is the foundation of language learning, particularly for one's second language (L2). The majority of vocabulary learning in L2 learners very often comes from explicit exposure and explicit teaching in the classroom (Ellis et al., 1994; Skehan, 1996; Willis, 1996; Ellis, 2000). However, explicit teaching cannot cover all the words that L2 learners need to master. A significant proportion of L2 words are acquired contextually. This is to say, L2 learners could learn novel words by extracting their meaning from linguistic context.

### The behavioral research on L2 contextual word learning

One controversial issue is how many encounters L2 learners need to acquire the meaning of a novel word (Nagy et al., 1987; Krashen, 1989; Pitts et al., 1989; Hulstijn et al., 1996). Some researchers believe that L2 learners need multiple times (Horst et al., 1998; Waring and Takaki, 2003; Tekmen and Daloglu, 2006; Webb, 2008; Pellicer-Sánchez and Schmitt, 2010). In Horst et al. study (1998), English as second language learners from Oman listen and read a simplified version of the novel, *The Mayor of Casterbridge*, to learn new words whose occurrence frequencies ranged from 2 to 17. Results showed that gaining the meaning of a new word needed at least eight exposures of that word. In Webb study (2007), English learners from Japan read sentences to learn new words, with the frequency of new words at one, three, seven, or ten. After reading, learners were tested on word form knowledge, morphological knowledge, and meaning. Results showed that the words were fully mastered when learned 10 times through reading. Waring and Takaki (2003) studied learners from Japan with lower L2 English proficiency. These learners were asked to read novels to learn new words, but these new words were pseudowords for an already known, very common concept, such as *windle* meaning “house.” Immediate testing after learning showed that these learners could master word form knowledge after reading the new words 8–10 times. However, even after they read novel words 15–18 times, they could not master the meaning of words. Based on these findings, researchers believed that to acquire the meaning of novel word needs more than 20 exposures. Here word form knowledge refers to the spelling of the word, and it is usually tested by asking participants to circle any words they could recognize from the text, as was used in Waring and Takaki (2003)'s study. Word meaning refers to the conceptual knowledge, and it is tested by asking participants to translate words into L1 (Waring and Takaki, 2003).

In sum, previous behavioral studies have shown that quantity of exposure, i.e., frequency of exposure, is important to L2 contextual word learning and that several repetitions are needed for learning to occur. However, what was left out in these studies was the sentence constraint effect. It has been found that learners learn fast in high constraint sentences. For example, in the study of Ma et al. (2015), Chinese learners of English were asked to read sentences with either high or low contextual constraint. Novel words (i.e., pseudowords) were embedded in these sentences. After reading, a pair of novel word and a real word with related or unrelated meaning was presented and the participants were asked to do a semantic relatedness judgment task. Results showed that the meaning of the novel words could be acquired in high constraint sentences but not in low constraint sentences, suggesting that high sentence constraint facilitates the acquisition of L2 contextual word meaning. This facilitation effect of high sentence constraint was also demonstrated in another study (Ma et al., 2016).

L2 contextual word learning was also influenced by proficiency level. Pulido (2003) recruited L2 learners with different proficiency levels and asked them to read narratives of familiar or less familiar topics in order to study L2 vocabulary acquisition and retention. These narratives contained non-sense words. Then, participants completed recognition tests 2 and 28 days after reading the narratives. The author found that no matter how familiar the topic was, learners with high proficiency acquired more words through reading and maintained their learning better. In the study by Tekmen and Daloglu (2006), Turkish learners of English at different proficiency levels read text to learn English words, and the results showed that higher proficiency readers acquired more words than lower level readers. In the study by Ma et al. (2015), adults with higher L2 proficiency performed better than lower-proficiency L2 learners in high constraint sentences. These findings demonstrate that higher proficiency levels could facilitate novel word learning.

### The event related potentials (ERP) research on L2 contextual word learning

Compared with behavioral studies, the ERP technique has high temporal resolution and could reveal ongoing brain responses of language processing. The amplitude of the N400 component measured at centroparietal electrodes is an index of the difficulty in integrating semantic information into context (Kutas and Hillyard, 1980; Holcomb and Neville, 1990; Nobre et al., 1994; Perfetti et al., 2005; Balass et al., 2010; Kutas and Federmeier, 2011). A larger N400 indicates more difficult semantic integration. In a similar sense, a decreasing N400 indicates the ease in processing. More recently, some researchers argue that the N400 is a measure of prediction in language processing (Federmeier et al., 2007, 2010; Brothers et al., 2015), with its amplitude being attenuated if the preceding context pre-activates the meaning of a word. The learning process of novel words could be revealed through changes in N400 amplitude (Mestres-Missé et al., 2007). Specifically, more frequent exposure to a novel word in a certain context would alleviate the difficulty in semantic integration, which could be reflected by a smaller N400.

Borovsky et al. (2010) adopted the ERP paradigm to explore the understanding and usage of L1 novel words learned through sentence reading. In their study, 26 English native speakers read high-constraint or low-constraint sentences with known words or unknown words(non-words, e.g., *marf*)embedded as the objects of transitive verbs in the test sentences, and then made a plausibility judgment of these words. The structure of the test sentence was always Pronoun-Transitive Verb—Article/Pronoun—Target word. Learners needed to determine if the word was used appropriately, e.g., they drove the *marf*. The plausibility effects could be reflected by a smaller N400 component in the appropriate condition than the inappropriate condition. The results showed the N400 component reduced only when the novel word was embedded in a high constraint sentence, suggesting that novel word usage could be rapidly acquired through high constraint sentences in native speakers.

Borovsky et al. (2012) further explored the factor of sentence constraint in the integration of novel word meanings into semantic memory using the same materials as in their 2010 study. Adult native speakers of English were asked to read high-constraint or low-constraint sentences that ended with known or unknown words. After reading, the participants did a lexical decision task to see whether the ending words (known or unknown) would show a priming effect on related, unrelated, and synonym target words. ERPs were also recorded during the experiment. The results showed that only when unknown words were embedded in high-constraint sentences, N400 amplitudes were different between related and unrelated target words, with unrelated targets eliciting the largest N400 and synonym targets eliciting the smallest N400. The results demonstrated that adult native speakers could rapidly integrate word meaning information into their mental lexicons by reading high constraint sentences.

Mestres-Missé et al. (2007) also observed real-time word meaning acquisition during sentence reading through amplitude changes in the N400. In this study, Spanish native speakers were asked to read three sentences with the same Spanish novel word. The meaning of the novel word was either consistent across the three sentences (congruent meaning, M+) or inconsistent (incongruent meaning, M−). The results showed that in the M+ condition, the N400 amplitude decreased across the three sentences, implying the acquisition of novel word meaning. Batterink and Neville (2011) used the same paradigm as Mestre-Missé, with pseudowords embedded in paragraphs. They also found decreased N400 amplitude as the number of sentences increased, but only in the congruent meaning condition. These studies indicate that for adult native speakers, when the quality of language input is high, word learning happens rapidly.

However, there have been few ERP studies investigating L2 contextual word learning. Elgort and Warren (2014) investigated the effect of L2 proficiency on L2 contextual word learning using the ERP technique. In this study, rare English words (i.e., critical words) were embedded in three high-constraint sentences. Participants read these sentences at their own pace. The following day, participants read these sentences again but with the critical words in the sentence-final position, followed by related or unrelated meaning probes. They were required to make semantic relatedness judgments about the critical words and the meaning probes, while ERPs were recorded. Results showed that for the higher proficiency group (students recruited in education and international business courses at the University of Pittsburgh), the N400 amplitude was significantly smaller in related trials than unrelated trials. However, this was not found in low proficiency learners (students recruited from English proficiency courses at the University of Pittsburgh, whose TOEFL iBT scores were below 100). These findings suggest that it is easier for learners with higher L2 proficiency to predict the meaning of rare words and form initial lexical semantic representations. Although Elgort and Warren (2014) examined the effect of L2 proficiency on L2 word learning, they did not record the change of brain responses as the novel words were being learned.

### The current study

It is still unknown how many exposures a learner needs for successful L2 contextual word learning. Few studies have manipulated the quality of sentence reading materials to study L2 contextual word learning. Variation of sentence constraint and/or comprehension difficulty of reading materials may lead to the unpredictability of L2 word learning times. Thus, we hypothesized that multiple times in L2 word learning are needed for low-constraint contexts. When reading materials were highly constrained (i.e., high quality), word learning can happen very rapidly. To verify this hypothesis, we used high-constraint sentences to explore the effect of number of exposure on L2 contextual word learning. Furthermore, L2 proficiency was also investigated in the current study.

We used the ERP technique, which has high temporal resolution, to explore the above questions. Following the design of Mestres-Missé et al. (2007), we embedded pseudowords at the end of the sentences, creating three conditions: pseudowords embedded in four consistent meaning sentences (M+ condition); pseudowords embedded in four inconsistent meaning sentences (M− condition); and a control condition with real words embedded in four consistent meaning sentences (R condition). Whereas, Mestres-Missé et al. (2007) focused on native speakers, the current study focused on L2 learners and the effect of their L2 proficiency.

Learners read four high-constraint sentences containing the same target word, and then judged the semantic relatedness between the target word and a meaning probe. The accuracy rates and response times were recorded to reveal their comprehension and acquisition of the meaning of novel words. Brain potential activities were recorded during sentence reading to observe the change of brain responses as the novel words were being learned.

In accordance with previous studies, we chose 300–500 ms post-stimulus as the time window to observe the N400 effect, which indicates the process of meaning acquisition (Perfetti et al., 2005; Mestres-Missé et al., 2007; Balass et al., 2010; Borovsky et al., 2010, 2012; Batterink and Neville, 2011) and meaning prediction in language processing (Federmeier et al., 2007, 2010; Brothers et al., 2015). For the behavioral data in the semantic relatedness judgment task, we predicted that there would be significant differences between the R and M- conditions, as well as between the M+ and M− conditions, but no difference between the R and M+ conditions. The proficiency of L2 may facilitate this learning process. For the ERP data, we predicted that the number of exposures would play a limited role in high-constraint sentences. More specifically, N400 amplitudes evoked by the last words of sentences in the R and M+ conditions would decrease as the number of sentences increased. As the same real word was used for the four sentences in the R condition, the N400 amplitude would keep unchanged across the second, third, and fourth readings since no effort is needed for meaning prediction. However, in the M+ condition, the meaning of the pseudoword is continually being predicted across the four sentences, so the N400 amplitude might keep decreasing following a downward slope. In the M− condition, no consistent meaning could be drawn from the four sentences, so the N400 amplitude would not change. Additionally, participants with higher English L2 proficiency might be fast learners in predicting the meaning of pseudowords, and thus would show a larger decrease of the N400 amplitude in the M+ condition.

## Methods

### Participants

Forty-four right-handed college students, who were all native Chinese speakers learning English as a second language, were recruited from Beijing Normal University. All participants had normal or corrected-to-normal vision. This study was approved by the ethics committee of the School of Psychology, Beijing Normal University. All participants gave their written informed consent before the experiment.

### Materials

#### Real words

Real words were 108 high frequency concrete nouns. Word frequency (mean logFreq = 10.04, *SD* = 0.95; logFreq refers to log-transformed HAL frequency norms) was rated according to HAL norms (Hyperspace Analog to Language Frequency Norms, Lund and Burgess, 1996; Balota et al., 2007). Concreteness (*M* = 578.37, *SD* = 42.54) was rated based on the MRC database (Medical Research Council Psycholinguistic Database, Wilson, 1988). Familiarity was rated using a 5-point scale (1 = very unfamiliar, 5 = very familiar) by a homogenous separate group of 26 college students from the same university (mean familiarity = 4.83, *SD* = 0.18).

#### Pseudowords

One hundred and eight pronounceable pseudowords were constructed using Wuggy (Keuleers and Brysbaert, 2010). Wuggy is a multilingual pseudoword generator using a specific algorithm to generate pseudowords which are matched with real English words in subsyllabic structure and transition frequency. We used real English words as base words to generate pseudowords (not the real words used as materials in Real Words). These real words have 2 or 3 syllables, and ranged from 5 to 7 letters in length. All pseudowords were randomly paired with real words as experiment materials.

Word length of pseudowords ranged from 5 to 7 (*M* = 5.69, *SD* = 0.57), and real words ranged from 3 to 10 (M = 5.13, *SD* = 1.54). There was a significant difference between the word length of pesudowords and their corresponding real words, *t* = 3.73, *p* < 0.01. Considering that word length difference may cause variances, we added word length (both pseudowords and real words) as a dummy predictor in the behavioral data analysis.

#### Semantically related/unrelated words

For the semantic relatedness judgment task, semantically related or unrelated words were selected to pair with the 108 real words. Word length of semantically related words ranged from 3 to 11 letters (*M* = 5.30, *SD* = 2.02), and semantically unrelated words ranged from 3 to 8 (*M* = 4.78, *SD* = 1.30). Word frequency was rated according to HAL norms (Hyperspace Analog to Language Frequency Norms, Lund and Burgess, 1996; Balota et al., 2007; semantic related words: mean logFreq = 9.74, *SD* = 1.42; semantic unrelated words: mean logFreq = 10.42, *SD* = 0.96). Semantic relatedness was rated by the 26 college students who rated the familiarity of real words (and who did not participate in the formal experiment), using a 5-point scale (1 = absolutely unrelated, 5 = closely related). For semantically related words, the average score was 4.43 (*SD* = 0.43), for example, “agriculture” was rated to be highly related to “farm” in meaning. For semantically unrelated words, the average score was 1.18 (*SD* = 0.21), for example, “bottle” was rated to be barely related to “mountain” in meaning.

#### Sentences

Four sentences were constructed for each real word, and then the real word was replaced by a pseudoword to create the M+ condition, in which the meanings of novel words were consistent and could be abstracted. The M− condition, in which the meanings of novel words were inconsistent and could not be abstracted, was created by reorganizing four sentences of four words into one group and replaced the four words with one pseudoword. The length of sentences ranged from 7 to 17 words, with the key word (real word or pseudoword) always appearing at the end of the sentence. The constraint of sentences was rated by a separate group of 43 college students from the same school of the participants. They completed the sentences in a cloze test with the first noun that came to their mind. The cloze probability was calculated by counting the percentage of times the same word was provided for each sentence. The mean cloze probability of these sentences was 89% (*SD* = 0.09). Rating through a 5-point scale (1 = very easy, 5 = very difficult), all sentences were easily understood (M = 1.45, *SD* = 0.19). And no statistical differences were found among the four sentences constructed for the same word [constraint: *F*_(3, 428)_ = 0.217, *p* = 0.89, Eta^2^ = 0.002; reading difficulty: *F*_(3, 428)_ = 1.01, *p* = 0.39, Eta^2^ = 0.007]. The sentences were split pseudo-randomly into three lists to make sure that no items repeated in one list, and the real words and their corresponding pseudowords never appeared in the same list. For each list, there were 576 sentences, 36 groups of experimental sentences per condition, and another 36 groups of filler sentences ending with real words. Each participant received only one of the three lists. Examples of a group of four sentences and test pairs of words from each condition are given in Table 1.

**Table 1:** Examples of four sentences and test pairs of words from each condition.

**Pseudoword**	**Real word**	**Semantic related/unrelated word**	**Semantic relationship**	**Four high constraint sentences that can form a congruent meaning (R/M+)**	**Four high constraint sentences that can not form a congruent meaning (M−)**
arram	Farm	Agriculture	Related	He raised chickens, sheep, and cows on the *farm/arram*	He raised chickens, sheep, and cows on the *arram*
				He knows well about planting crops because he lives on the *farm/arram*	A good salesman should win the trust of the *arram*
				Cotton, corn and vegetables were all grown on his *farm/arram*	The boy fell in love with a girl, and he wrote her a love *arram*
				Kids of poor village families often help parents with the work on the *farm/arram*	They made fun of me by putting salt in my coffee instead of *arram*
banble	Shape	Hotel	Unrelated	Circles, triangles, and squares are different in *shape/banble*	Circles, triangles, and squares are different in *banble*
				Liquid flows freely without a fixed *shape/banble*	They played in the river, and caught several *banble*
				Blind person use their fingers to feel the object's *shape/banble*	To guard the house against thieves, they raised a *banble*
				The building looks like a ball, it's round in *shape/banble*	No one answered the door, when I rang the *banble*

#### English level evaluation tool

The College English Test (CET) (see the Procedure section) and Quick Placement Test (2001) was used in this experiment to evaluate the participants' English level. The QPT is a flexible test developed by Oxford University Press and Cambridge ESOL to quickly evaluate a student's level of English. It includes reading and structure, grammar, and vocabulary. Part 1 has 40 items and Part 2 has 20 items, for a maximum score of 60. The scores of QPT are presented in Table 2.

**Table 2:** Background information of participants by proficiency level: Mean (*SD*).

**Participants**	**Age**	**AoA**	**Self-rating English Level**				**QPT score**
			**Listening**	**Speaking**	**Reading**	**Writing**	
Higher proficiency	21.63	10.67	3.21	3.13	3.96	3.66	48.67
	(1.69)	(1.09)	(0.58)	(0.34)	(0.55)	(0.64)	(3.19)
Lower proficiency	22.15	11.30	2.50	2.40	3.30	3.05	40.90
	(2.99)	(1.03)	(0.89)	(0.82)	(0.66)	(0.60)	(3.86)
*t* test	−0.73	−1.97	3.16^*^	3.95^**^	3.62^*^	3.27^*^	7.31^**^

AoA, English Age of Acquisition;

* p < 0.01;

** *p < 0.001*.

### Experimental design

This study used a mixed experimental design: 4 (sentence presentation order: 1st, 2nd, 3rd, 4th) × 3 (word type: R, M+, M−) × 2 (proficiency level: higher, lower). Here, sentence presentation order and word type were within-subjects factors, and proficiency was a between-subjects factor. All the sentences were counter-balanced across participants according to word type (R, M+, M−), to make sure no words/pseudowords or sentences were repeatedly presented for each participant. The four sentences within each group were presented randomly.

### Procedure

Participants were divided into two groups based on their College English Test (CET) levels. The CET is a test designed by the Ministry of Education of China to estimate the English proficiency level of Chinese college students. It includes listening comprehension, reading comprehension, writing, translation, and cloze task (Zheng and Cheng, 2008). Twenty-four participants who passed CET Band 6 were categorized as higher proficiency English learners; 20 participants^1^ who failed CET Band 4 were categorized as lower proficiency English learners. Before the experiment, all participants completed self-ratings of their English listening, speaking, reading, and writing abilities on a 5-point scale (1 = very non-proficient, 5 = very proficient) as well as the QPT. For details about participants' scores, see Table 2.

E-prime software version 2.0 was used to present stimuli on a computer screen. Participants were seated in front of the computer and practiced several trials prior to the formal experiment.

The first part of each sentence was presented as a whole, with the last word of each sentence presented separately. The experiment began with the presentation of a fixation cross in the center of the screen for 500 ms. After the fixation, the first part of the sentence was presented and would not disappear until learners pressed the spacebar to continue, and then a blank screen lasted 1,000 ms, followed by the last word/pseudoword of the sentence which was presented for 500 ms, and then a blank screen lasted for 1,200 ms followed by the next trial started.

Each group included four sentences of one word/pseudoword and each block included six groups of sentences. When learners finished a block, a question mark appeared on the screen for 1,000 ms as a prompt for participants to do the semantic relatedness judgment. In this task, learners read six word pairs corresponding to the six groups of sentences just presented and judged whether the words were semantically related. Within each block, the six groups of sentences and the corresponding word pairs were presented in pseudo-random order. “Related” or “Unrelated” responses were made by pressing “F” or “J” on the keyboard. Half of the participants were asked to press “F” for “Related,” “J” for “Unrelated.” The other half press “J” for “Related,” “F” for “Unrelated.” The two words appeared on the screen simultaneously, and if no response was detected within 5,000 ms, the stimuli would disappear followed by a blank screen for 200 ms. The whole experiment lasted for 1.5–2 h.

Finally, all participants were given a checklist of all the sentences they had just read to confirm that they had no difficulty in reading these sentences. In this checklist, all the pseudowords were replaced with the corresponding real words. The participants were asked to mark the sentences or words which were difficult to them. Because no marks were made on any items, we presume the materials could be easily processed by the participants.

### Behavioral analysis

The behavioral data for all 44 participant learners were reviewed. Cases of no response or responding too early (less than 200 ms) were excluded (1.66%). A mixed-effects logistic model of accuracy and a mixed-effects model of response time were built to analyze their performance in the semantic relatedness judgment task (Baayen et al., 2008; Jaeger, 2008). These two models were built with subjects and items as random intercepts and slopes. All statistical analyses were carried out using R 3.1.2 (R Core Team, 2014), implemented with package lme4 (Bates et al., 2013), lmerTest (Kuznetsova et al., 2013), and multcomp (Hothorn et al., 2008).

### EEG recording and analysis

Participants were seated comfortably in a chair, relaxing, and minimizing eye movements and blinks. They read the sentences quietly. The electroencephalogram (EEG) was recorded from 64 Ag/AgCl electrodes placed according to the extended 10–20 positioning NeuroScan 4.5 system (http://www.neuroscan.com/). The signal was recorded with 500 Hz sampling rate and referenced online to the right mastoid (M2). Electrode impedances were kept below 5 kΩ. The EEG activity was filtered online within a bandpass of 0.05–100 Hz and later low-pass (30 Hz) refiltered offline. Eye blinks were mathematically corrected according to the recorded VEOG and HEOG. This mathematical algorithm is a regression analysis in combination with artifact averaging to produce a reliable and valid method for artifact removal (Semlitsch et al., 1986). The remaining artifacts were manually rejected. The EEG signal was recorded during the entire experiment, including the sentence reading and semantic relatedness judgment tasks.

Segments with electrical activity ±100 μV at any electrode sites were rejected. EEG segments of 800 ms with a pre-stimulus (the last word of the sentence) baseline time of 100 ms were selected and averaged offline to obtain the ERPs. Baseline correction was performed in relation to the pre-stimulus time. The signals were re-referenced using an average value of both right and left mastoid offline.

One lower proficiency learner was excluded due to too many artifacts (only 33.33% of trials were available), so the final dataset was 43 participants, including 24 higher proficiency learners and 19 lower proficiency learners. After artifact rejection, 5.3% of trials were rejected.

In accordance with previous studies, we analyzed the mean amplitude within the time window of 300–500 ms upon the presentation of the last word of the sentence. To increase the signal-to-noise ratio over the 64 channels, as done in the study by Batterink and Neville (2013), we focused on 36 electrodes across 6 scalp areas: left-anterior (AF3, F3, F5, F7, FC3, FC5), left-posterior (CP3, CP5, P3, P5, P7, PO3), right-anterior (AF4, F4, F6, F8, FC4, FC6), right-posterior (CP4, CP6, P4, P6, P8, PO4), central-anterior (F1, Fz, F2, FC1, FCz, FC2), central-posterior (CP1, CPz, CP2, P1, Pz, P2). A five-way repeated-measures ANOVA with the factors sentence presentation order (first, second, third, fourth), word type (R, M+, M−), proficiency (higher, lower), hemisphere (left, central, right), and brain region (anterior, posterior) was applied on the mean amplitudes. The Greenhouse-Geisser correction was applied on all *p*-values of main effects and interactions.

## Results

### Behavioral data results

For each of the two proficiency groups, accuracy and response times for different conditions are shown in Table 3.

**Table 3:** Accuracy (%) and response time (ms) of semantic relatedness judgment task: Mean (*SD*).

	**R**	**M+**	**M−**
**HIGHER PROFICIENCY**			
Accuracy	90 (29)	87 (34)	69 (46)
Response time	1,462 (739)	1,475 (721)	1,855 (835)
**LOWER PROFICIENCY**			
Accuracy	85 (36)	86 (35)	63 (48)
Response time	1,539 (752)	1,583 (761)	1,879 (845)

A mixed-effects logistic model of accuracy was built in which word type and proficiency were fixed factors, subject and item (i.e., combination of sentences and key words) were random factors, and word length was a covariant (Table 4). A Tukey *post-hoc* test was applied to reveal the simple effects of word type. Results were summarized in Table 5. As we can see from Tables 4, 5, there was a significant main effect of word type, such that accuracy was higher in the R condition than in the M− condition (*z* = 9.68, *p* < 0.001), higher in the M+ than in the M− condition (*z* = 8.02, *p* < 0.001), but no significant difference between the R condition and the M+ condition was observed (*z* = 1.07, *p* = 0.655).Word length did not affect the main effects of fixed and random factors.

**Table 4:** Mixed-effects logistic model of accuracy in the semantic relatedness judgment task.

**Predictor**	**Estimate**	**SE**	***z*-value**	***p* (>|z|)**
(Intercept)	0.4406	0.4874	0.90	0.366
Word Type M+	1.4099	0.1457	9.68	0.000
Word Type R	1.6444	0.2051	8.02	0.000
Proficiency	0.0561	0.2658	0.21	0.833
Word Length	0.0638	0.0767	0.83	0.405
Word Type M+: lower proficiency	0.6290	0.2164	2.91	0.004
Word Type R: lower proficiency	−0.6072	0.2227	2.73	0.006

**Table 5:** Tukey *post-hoc* test of accuracy in semantic relatedness judgment task.

**Linear hypotheses**	**Estimate**	**SE**	***z* value**	***p* (>|z|)**
M+ - M− = 0	1.4099	0.1457	9.68	0.000
R - M− = 0	1.6444	0.2051	8.02	0.000
R - M+ = 0	0.2344	0.2184	1.07	0.655
Lower proficiency-Higher proficiency = 0	0.0561	0.2658	0.21	0.996
Higher proficiency (R - M+) = 0	0.2344	0.2184	1.07	0.869
Higher proficiency (R - M−) = 0	1.6444	0.2051	8.02	0.000
Higher proficiency (M+ - M−) = 0	1.4099	0.1457	9.68	0.000
Lower proficiency (R - M+) = 0	0.2126	0.2380	0.89	0.935
Lower proficiency (R - M−) = 0	2.2516	0.2189	10.29	0.000
Lower proficiency (M+ - M−) = 0	2.0390	0.1639	12.44	0.000
R(Lower proficiency-Higher proficiency) = 0	0.6633	0.3022	2.20	0.208
M+(Lower proficiency-Higher proficiency) = 0	0.6851	0.2956	2.32	0.161
M−(Lower proficiency-Higher proficiency) = 0	0.0561	0.2658	0.21	0.999

We also examined whether the accuracy of different conditions was significantly higher than chance level. For the R condition, accuracy was above chance level (higher proficiency learners: *z* = 3.92, *p* < 0.01; lower proficiency learners: *z* = 3.13, *p* < 0.01); for the M+ condition, accuracy was also above chance level (higher proficiency learners: *z* = 3.63, *p* < 0.01; lower proficiency learners: *z* = 3.22, *p* < 0.01); for the M- condition, however, there was no significant difference between accuracy and chance level (higher proficiency learners: *z* = 1.86, *p* > 0.05; lower proficiency learners: *z* = 1.16, *p* > 0.05).

For the response time data, a mixed-effects model was constructed in which word type and proficiency were fixed factors, subject and item (combination of sentences and key words) were random factors, and word length was a covariant. Results are summarized in Table 6. A Tukey *post-hoc* test was conducted to reveal the simple effects of word type and proficiency. Results are summarized in Table 7. There was a significant main effect of word type, such that response time was shorter in the R condition than in the M− condition (*z* = −10.43, *p* < 0.001), shorter in the M+ condition than in the M− condition (*z* = −12.77, *p* < 0.001), but no significant difference between the R condition and the M+ condition was observed (*z* = −0.026, *p* = 1.000). And there was also a significant main effect of proficiency, such that higher proficiency learners responded faster than lower proficiency learners (*z* = 7.75, *p* < 0.001). Word length did not affect the main effects of fixed and random factors.

**Table 6:** Mixed-effects model of response time in semantic relatedness judgment task.

**Predictor**	**Estimate**	**SE**	***t* value**	***p*(>|t|)**
(Intercept)	1484.67	117.57	12.63	0.000
Word Type M+	−384.49	30.11	−12.77	0.000
Word Type R	−385.45	36.95	−10.43	0.000
Proficiency	621.24	80.14	7.75	0.000
Word Length	19.50	12.65	1.54	0.124
Word Type M+: Lower Proficiency	79.84	45.03	1.77	0.076
Word Type R: Lower Proficiency	48.02	45.01	1.07	0.286

**Table 7:** Tukey *post-hoc* test of response time in semantic relatedness judgment task.

**Linear hypotheses**	**Estimate**	**SE**	***t* value**	***p*(>|t|)**
M+ - M− = 0	−384.49	30.11	−12.77	0.000
R - M− = 0	−385.45	36.95	−10.43	0.000
R - M+ = 0	−0.97	36.83	−0.03	1.000
Lower proficiency-Higher proficiency = 0	621.24	80.14	7.75	0.000

### EEG data results

**Figure 1:**
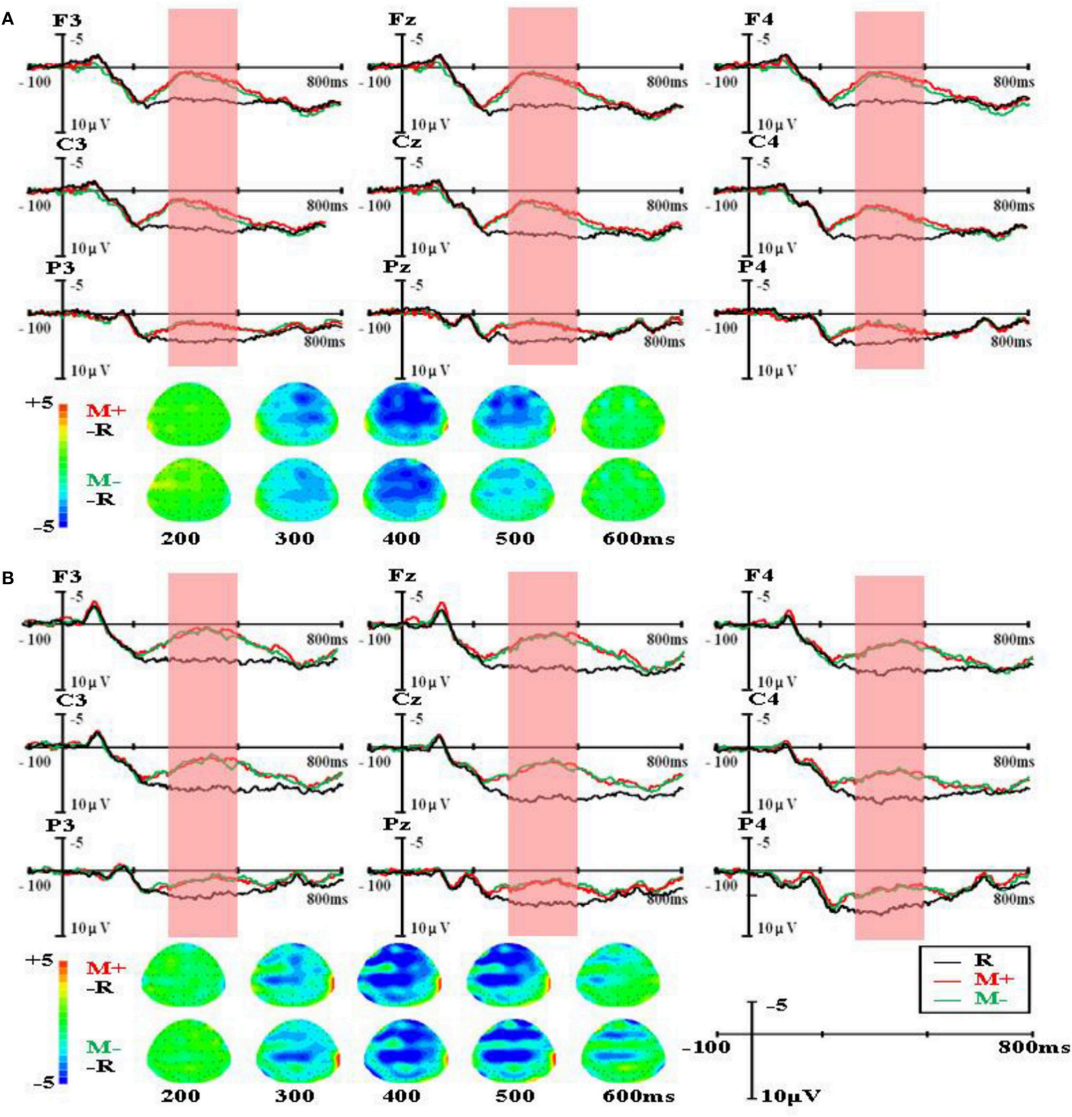
The group-level average waveforms and scalp distribution elicited by different word types of the first presented sentences for higher proficiency **(A)** and lower proficiency participants **(B)**.

**Figure 2:**
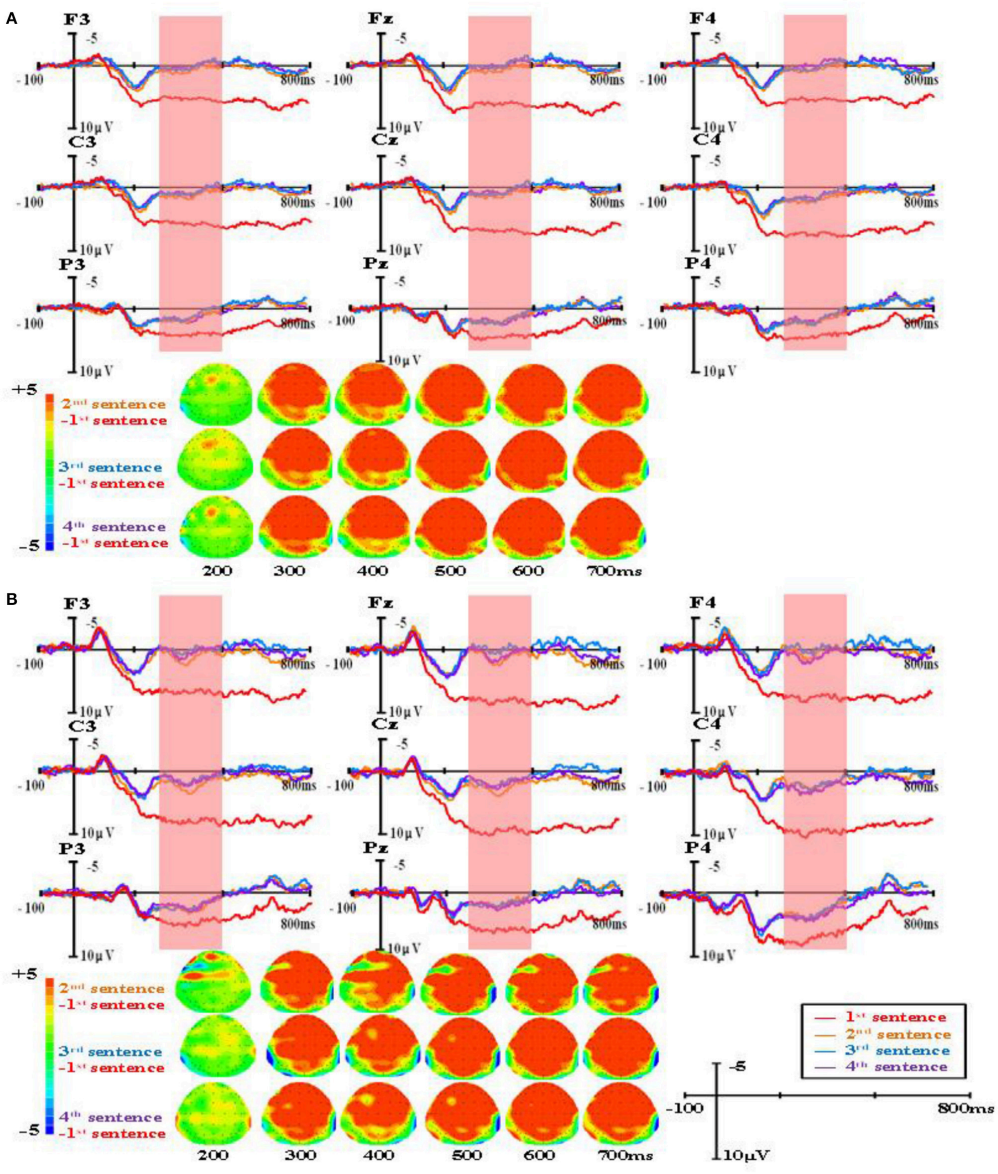
The group-level average waveforms and scalp distribution elicited by real words of four sentences (R condition) for higher proficiency **(A)** and lower proficiency participants **(B)**.

**Figure 3:**
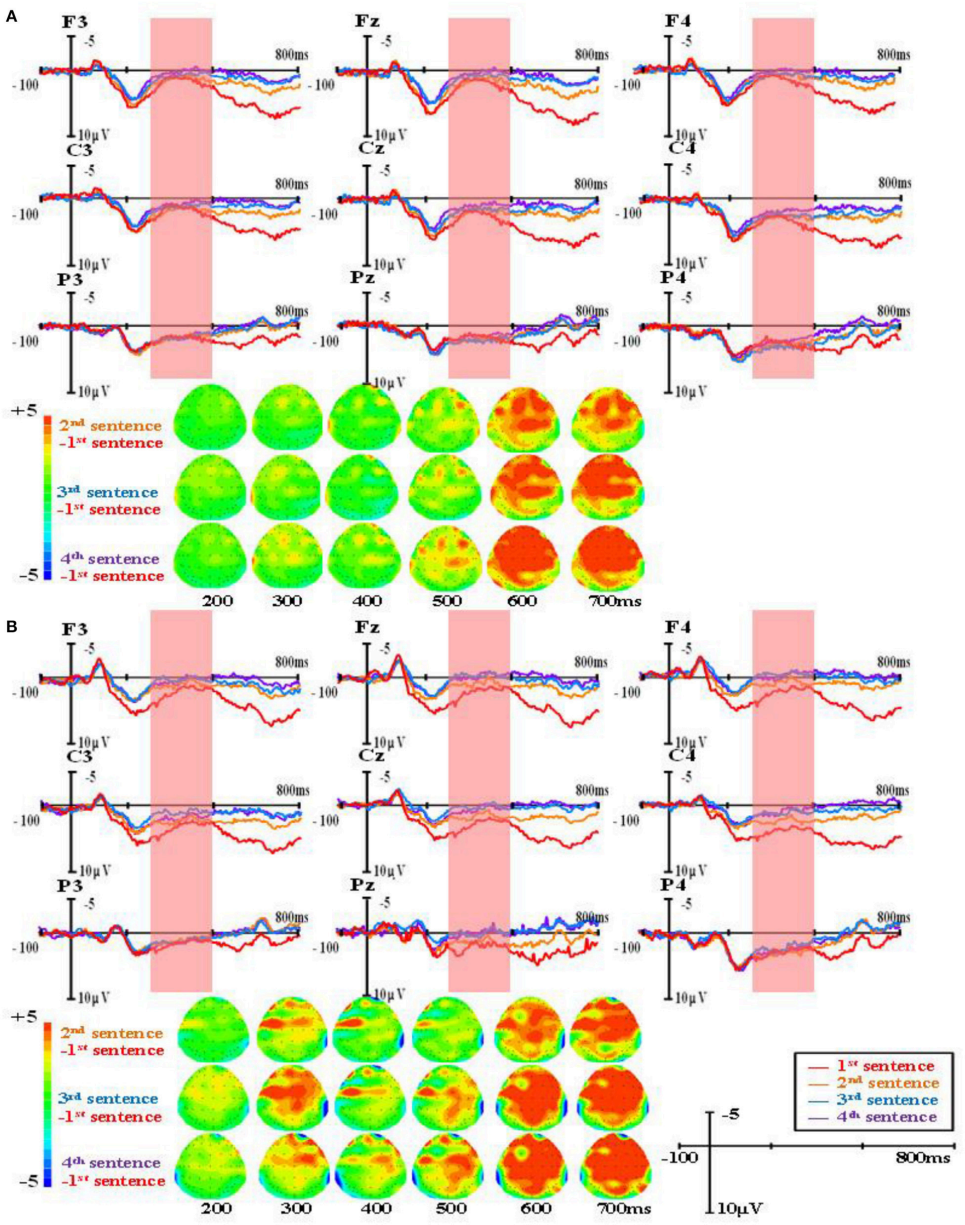
The group-level average waveforms and scalp distribution elicited by pseudowords of four sentences (M+ condition) for higher proficiency **(A)** and lower proficiency participants **(B)**.

**Figure 4:**
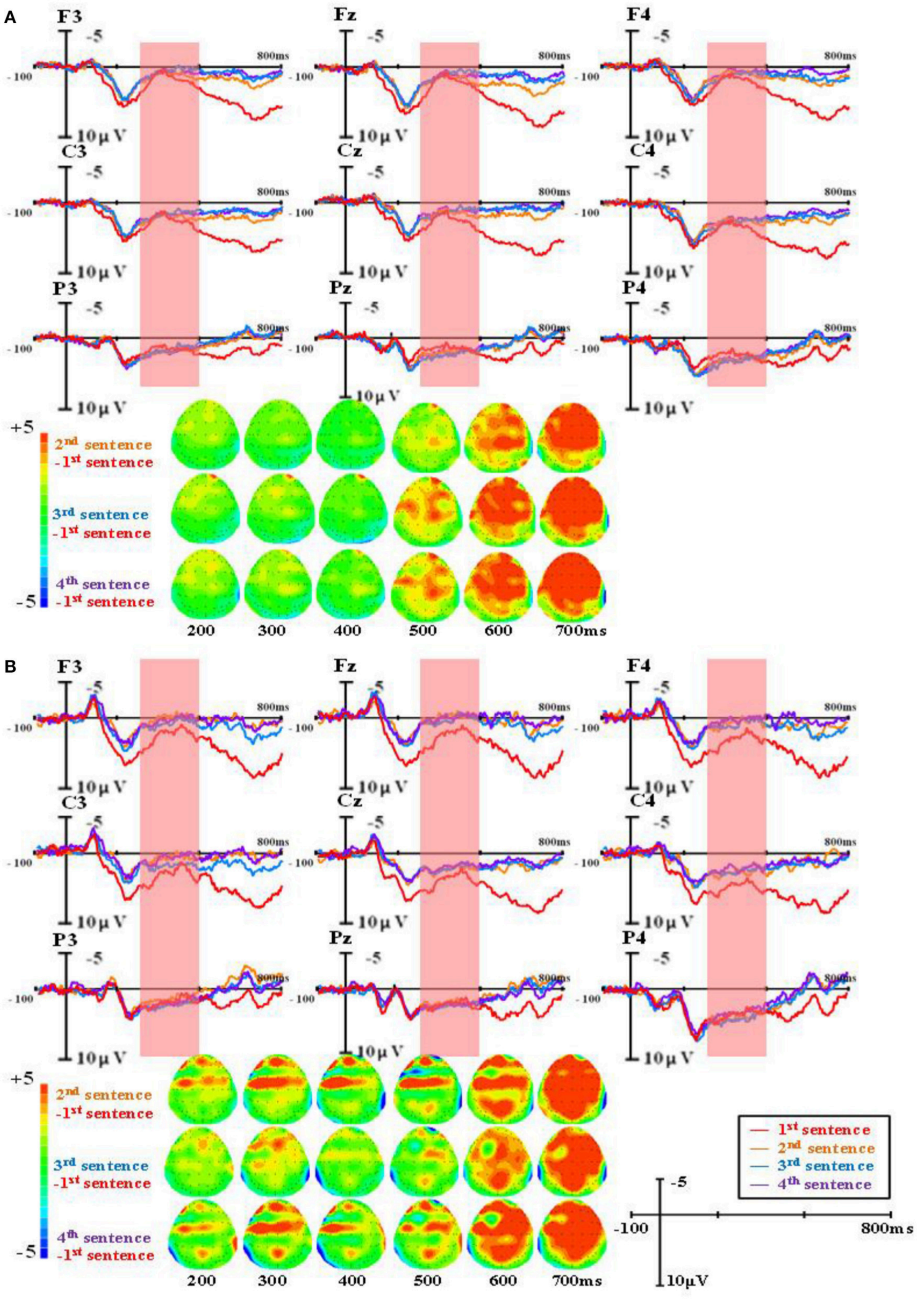
The group-level average waveforms and scalp distribution elicited by pseudowords of different sentences (M- condition) for higher proficiency **(A)** and lower proficiency participants **(B)**.

The group-level average waveforms and scalp distribution elicited by different word types are shown in Figure 1 (the first presented sentences), Figure 2 (the four sentences in the R condition), Figure 3 (the four sentences in the M+ condition), and Figure 4 (the four sentences in the M− condition). We chose the 300–500 ms post-stimulus time window (upon the presentation of the last word of the sentence) to analyze the mean amplitudes. From the waveforms and scalp distribution, the M+ and M− conditions evoked obvious negative components in the first presented sentences, indicating difficulty in semantic integration. (Figure 1A for higher proficiency learners, Figure 1B for lower proficiency learners).

A repeated-measures ANOVA was conducted on the mean amplitude in the 300–500 ms time window, and the results showed a significant main effect of word type, *F*_(2, 82)_ = 4.90, *p* < 0.05, Eta^2^ = 0.11. There was also a significant main effect of sentence presentation order, *F*_(3, 123)_ = 28.73, *p* < 0.001, Eta^2^ = 0.41. More importantly, there was a significant interaction between word type and sentence presentation order, *F*_(6, 246)_ = 12.57, *p* < 0.001, Eta^2^ = 0.24. Further analysis found that in the R condition, N400 amplitudes evoked in the first presented sentences were significantly larger than in the second (MD = 3.032, SE = 0.309, *p* < 0.001), the third (MD = 3.086, SE = 0.306, *p* < 0.001), and the fourth (MD = 3.210, SE = 0.327, *p* < 0.001). No difference was found among the second, third, and fourth sentences. In the M+ condition, N400 amplitudes evoked in the first presented sentences were significantly larger than in the third (MD = 1.149, SE = 0.342, *p* < 0.05) and the fourth presented sentences (MD = 1.291, SE = 0.328, *p* < 0.01). There were no differences between the first and second sentences or among the second, third, and fourth sentences. In the M− condition, no sentence presentation order effect was found.

There was a significant main effect of brain region, *F*_(1, 41)_ = 12.76, *p* < 0.01, Eta^2^ = 0.24, such that N400 amplitudes evoked in the anterior were significantly smaller than in the posterior (MD = −0.937, SE = 0.267, *p* < 0.01). There was a significant main effect of hemisphere, *F*_(2, 82)_ = 22.34, *p* < 0.001, Eta^2^ = 0.35, such that N400 amplitudes evoked in the left hemisphere were significantly smaller than in the central midline (MD = −0.715, SE = 0.122, *p* < 0.001) and right hemisphere sites (MD = −0.947, SE = 0.148, *p* < 0.001), while no difference was found between the central midline and right hemisphere sites (MD = −0.232, SE = 0.145, *p* = 0.351).

There was a significant interaction between sentence presentation order and brain region, *F*_(3, 123)_ = 15.91, *p* < 0.001, Eta^2^ = 0.28. Further analysis found no brain region effect on the first presented sentence (MD = −0.029, SE = 0.248, *p* = 0.919), while significant brain region effects in the second (MD = −0.978, SE = 0.290, *p* < 0.01), third (MD = −1.334, SE = 0.327, *p* < 0.001), and fourth presented sentences (MD = −1.404, SE = 0.316, *p* < 0.001) were observed, exhibiting more negative EEG signals at anterior sites than at posterior sites.

There was a significant three-way interaction among word type, brain region, and proficiency, *F*_(2, 82)_ = 5.27, *p* < 0.01, Eta^2^ = 0.17. Further analysis found significant brain region effects for higher proficiency learners in all three conditions: R (MD = −1.072, SE = 0.347, *p* < 0.01), M+ condition (MD = −1.282, SE = 0.389, *p* < 0.01), and M− (MD = −0.769, SE = 0.378, *p* = 0.048), exhibiting smaller N400 amplitude at anterior sites than at posterior sites. In lower proficiency learners, significant brain region effects were only found in the M- condition (MD = −1.114, SE = 0.425, *p* < 0.05), but not in the R (MD = −0.638, SE = 0.390, *p* = 0.11) and M+ conditions (MD = −0.744, SE = 0.437, *p* = 0.10).

The main findings was that in the R condition, N400 amplitudes evoked in first presented sentences were significantly larger than in the second, third, and fourth presented sentences. In the M+ condition, N400 amplitudes evoked in the first presented sentences were significantly larger than in the third and the fourth presented sentences, suggesting that participants gradually acquired the meaning of the pseudoword throughout sentence reading. In the M− condition, no sentence presentation order effect was found.

## Discussion

The present study explored the effects of high quality sentence encounters and proficiency level on L2 contextual word learning. Behavioral results of accuracy showed no statistical difference between the M+ condition and the R condition for both groups of learners, but lower accuracy in the M− condition. These results suggests that the M+ condition—but not the M− condition—effectively facilitates novel word meaning acquisition. Besides, accuracy results showed that both groups of learners were equally familiar with the real words, and they both acquired the novel word meaning in the M+ condition. However, response time results showed that higher proficiency learners spent less time in the semantic relatedness judgment task, suggesting that higher proficiency learners were better at processing novel words as well as already known words.

For the ERP results, in 300–500 ms time window, significant negative components were found in the M+ and M− conditions compared to the R condition when learners read the first sentence. This negative component normally found between 300 and 500 ms in the frontal and parietal regions was evoked by semantic violation, and it is a typical N400 effect in sentence reading. As the sentence number increased, the N400 patterns for the three types of words started to differ. In the R condition, the N400 amplitude decreased rapidly; in the M+ condition, the N400 amplitude decreased slowly and this decrease became significant upon the third sentence; in the M− condition, the N400 amplitude showed no obvious changes across the four sentences. Consistent with our predictions, this divergence in N400 amplitude change among the three conditions directly reflected the course of word learning. Real words are words learners already know and don't need to be learned again. Novel words in the M+ condition are words for which learners could form consistent meanings, so they can be learned gradually as sentence number increases, and this learning process could be reflected directly by the decreasing of the N400 amplitude. Novel words in the M− condition are words for which learners could not form consistent meanings, so they cannot be learned and no changes would be observed in the EEG signal.

According to previous findings, L2 word learning through sentences needs multiple exposures. However, previous studies did not control sentence quality, and have not explored how sentence quality modulates the influence of exposure times. In this study, the EEG signals showed novel words were learned successfully in the M+ condition as sentence number increased, suggesting that the first two high quality sentences might play an important role in providing multiple exposures. We believe the key point between few encounters or multiple encounters needed to acquire L2 word meaning is the quality of language input. When the quality of language input is low, more exposures are needed. When the quality of language input is high, L2 learners could rapidly assign meaning to the novel word. This is to say, when the sentence contexts are highly constrained, the number of exposures of L2 novel words does not matter that much. It is the high quality that really matters.

The current findings are consistent with previous studies on L1 contextual word learning. Borovsky et al. (2010, 2012) found that native speakers could rapidly acquire the meaning of unknown words in strongly constrained sentences. Mestres-Missé et al. (2007) recorded ERP signals upon novel words during sentence reading, and found that with highly constrained sentences, participants were able to predict the meaning of novel words at the second presented sentence and fully understand the novel word at the third sentence. The current study also found that L2 learners could learn the meaning of novel words rapidly in highly constrained sentences. These consistent findings from native speakers and L2 learners suggest that high constraint facilitates contextual word learning. What the current study contributes to the L2 literature is that the change of brain responses upon novel word exposures was recorded, revealing the online process of L2 contextual word learning.

Contrary to one prediction, we did not observe the effect of L2 proficiency in the semantic relatedness judgment accuracy and the N400 amplitude. Nevertheless, higher proficiency learners did respond faster than lower proficiency learners in the semantic judgment task. The possible reasons for this weak L2 proficiency effect might be, firstly, that the materials used in this study might be too easy for all the participants; second, the difference between the higher proficiency level and lower proficiency level might not be large enough. Nonetheless, considering previous findings about the effect of language proficiency in native speakers (Perfetti et al., 2005; Balass et al., 2010) and L2 learners (Pulido, 2003; Tekmen and Daloglu, 2006; Ma et al., 2015), we believe that language proficiency is an important factor in L2 contextual word learning. Learners with higher proficiency could be better able to learn novel words because they have already accumulated sufficient word knowledge.

Here it should be noted that in the present study, we only focused on the very initial phase of word meaning acquisition, namely, the process of building form-meaning mapping, but not on the succeeding consolidation phase. To reach the final goal of word acquisition, more exposures are needed for the consolidation of form-meaning mappings.

## Conclusion

In sum, by creating four high quality sentences for each novel word, and recording the brain electrical activity during word learning through reading, we directly observed real-time L2 contextual word learning. The results provide direct evidence that L2 learners can rapidly acquire word meaning in high constraint sentences without multiple times of exposure, and L2 proficiency level affects learners' efficiency of using high quality language information.

## Author contributions

TM and BC designed the experiment and wrote the manuscript; TM collected and performed data analysis; LL and HL edited and revised the manuscript.

### Conflict of interest statement

The authors declare that the research was conducted in the absence of any commercial or financial relationships that could be construed as a potential conflict of interest. The reviewer, LM, and handling Editor declared their shared affiliation.

## Footnotes

^1^Due to the longer duration (1.5–2 h) of the experiment, we were not able to recruit an equal number of high- and low-proficiency English learners. Hopefully, the mixed-effect model analysis would guarantee the validity of the behavioral results despite uneven sample sizes (Lan et al., 2015).
